# What can MaxEnt reveal about high-density recordings and what can high-density recordings reveal about MaxEnt?

**DOI:** 10.1186/1471-2202-12-S1-P146

**Published:** 2011-07-18

**Authors:** Dagmara Panas, Alessandro Maccione, Luca Berdondini, Matthias H Hennig

**Affiliations:** 1 Institute for Adaptive and Neural Computation, School of Informatics, University of Edinburgh, EH8 9AB, UK; 2 Department of Neuroscience and Brain Technologies, Italian Institute of Technology, 16163 Genova, Italy

## 

Recent advances in neural recording techniques open exciting possibilities of better understanding whole populations of neurons. Devices such as APS MEA (Active Pixel Sensor Microelectrode Array) [1,2] allow for simultaneous recordings from 4096 channels (64x64 grid) at near-cellular resolution (electrode size: 21μm, electrode spacing: 42μm) and constitute a potentially very rich and detailed source of information on the dynamics of neural systems. Such volumes of data are however difficult to analyse: simple measures such as mean firing rates and correlations are often insufficient to capture interesting phenomena, while more sophisticated approaches can be computationally intensive and hard to interpret. Here we examine the applicability of pairwise maximum entropy (MaxEnt) [3-5] modelling to describe APS MEA data.

Pairwise maximum entropy model (equivalent to Ising model in physics), when fit to the data, yields a minimally structured probability distribution of network states that respects first and second order interactions. It is a convex, parsimonious and readily interpretable model that has been shown to characterize spiking patterns surprisingly robustly in many cases [3,4]. Additionally, it can provide a sensitive tool in detecting higher-order interactions. As reported in [5], the significant failure of the Ising model in close range (<300 μm) uncovers a high-order processing mode in local clusters of neurons, a mode of processing absent on larger scale (>600 μm) and undetectable with correlations.

**Figure 1:**
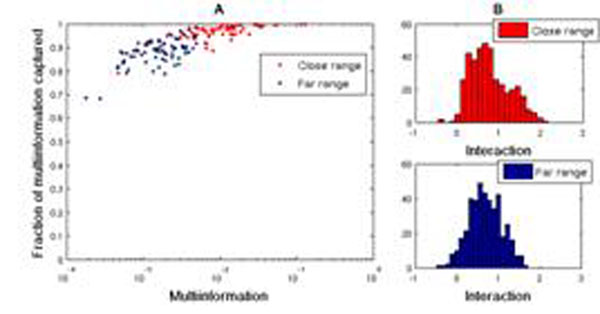
**A** Fraction of multiinformation captured by the MaxEnt model versus multiinformation (bits per bin, 5ms bins used). **B** Histograms of the values of fitted interaction parameters.

In present work we examine the results and performance of the MaxEnt model fitting in different preparation types and parameter regimes; owing to high resolution recordings we can specifically focus on varying spatial scales. As can be seen in Fig.1, indeed even in cultured tissue data there are indicators of certain discrepancies between local populations and populations further apart. Firstly (panel A), it is in local populations where the advantage of Ising model over the independent model is most prominent. Secondly (panel B), the interactions within local populations reveal a different structure than those among groups of neurons spread further apart (Kolmogorov-Smirnov test, p<0.05); and, importantly, this is not a feature that can be shown by correlation analysis.
